# Pre-resection Meningioma Embolization Does Not Improve Time to Recurrence in a Heterogeneous Cohort: A Retrospective Propensity-Matched Cohort

**DOI:** 10.7759/cureus.95173

**Published:** 2025-10-22

**Authors:** Anthony V Nguyen, Buqing Liang, Walter S Lesley, Frank S Harris, Ethan A Benardete, James K Cooper, David Garrett, Dongxia Feng, Ibrahim M Elnihum, Jason H Huang

**Affiliations:** 1 Department of Neurosurgery, Baylor Scott & White Medical Center - Temple, Temple, USA; 2 Department of Neurosurgery, Loma Linda University Medical Center, Loma Linda, USA; 3 College of Medicine, Texas A&M University, Bryan, USA; 4 Department of Medicine, Baylor College of Medicine, Temple, USA

**Keywords:** central nervous system neoplasms, embolization, endovascular procedures, meningioma, neurosurgery

## Abstract

Introduction

Meningiomas are the most common primary tumors of the central nervous system, and pre-resection meningioma embolization has been touted to reduce surgical blood loss; however, high-quality evidence supporting its routine employment is lacking. A recent study demonstrated that embolization benefited progression-free recurrence, so we aimed to evaluate the safety and efficacy of pre-resection meningioma embosurgery.

Methods

This retrospective cohort study examined adult patients who underwent intracranial meningioma resection from January 2014 to July 2019. One-to-one propensity score matching was applied. The association of embolization with operative time, estimated blood loss (EBL), hemoglobin drop, length of stay, need for blood transfusion, achievement of gross total resection, complication incidence, death within 90 days, tumor recurrence, and time to recurrence was analyzed.

Results

Of 116 patients meeting study criteria, 32 underwent embolization (28.6%). After excluding World Health Organization grade 2-3 meningiomas and propensity score matching, there were 28 patients in each of the embolization and non-embolization groups. Embolization was not associated with operative time, EBL, post-resection hemoglobin drop, perioperative transfusions, complications, or death. Embolization was associated with increased post-resection length of stay (9.9 + 1.3 days versus 6.6 + 1.3 days, P = 0.002). In survival analysis, embolization was not associated with improved recurrence-free survival.

Conclusions

While meningioma embolization appears safe, its long-term efficacy is unclear. Embosurgery was not associated with increased time to recurrence in this patient cohort, but the observed results may be due to differences in technique, tumor genomics, or patient selection. Prospective, randomized studies are warranted for further investigation.

## Introduction

Meningiomas are the most prevalent primary tumors of the central nervous system [1]. The majority of meningiomas are benign, and surgery is often indicated for symptomatic lesions [2]. Given that meningiomas are generally benign, attempting gross total resection is justified. However, as a central nervous system tumor, these are often intertwined with critical neurovascular elements that must not be violated. As meningiomas are extra-axial tumors arising from arachnoid cap cells and seldom invade the brain parenchyma, the goal of surgery is to minimize any damage to neural structures. The general technique for meningioma resection is sometimes referred to as the "4 Ds," which consist of dissection, detachment, devascularization, and debulking [3]. One technique for accomplishing tumor devascularization preoperatively is embolization.

Preoperative meningioma embolization was first described by Manelfe et al. in the French literature in 1973 [4]. A number of studies continue to support meningioma embosurgery as a useful neoadjuvant treatment with benefits ranging from decreased blood loss to increased time to recurrence [5-9]. However, others have noted that embolization is not without risk, and there is still some controversy surrounding its employment in practice [7,9-11]. These studies draw unclear conclusions regarding the effects of embolization on blood loss and surgical morbidity while reporting non-zero complication rates [9-11]. In light of the recent findings of prolonged time to recurrence of embolized meningiomas by Akimoto et al., we sought to investigate whether the published benefits (decreased blood loss, lower surgical morbidity, and increased time to recurrence) of pre-resection meningioma embosurgery were reproducible in our institutional cohort [6]. This study follows the Strengthening the Reporting of Observational Studies in Epidemiology (STROBE) guidelines.

## Materials and methods

This was a retrospective cohort study of all patients aged >18 years with intracranial meningiomas who underwent surgical resection consecutively between January 2014 and July 2019. Follow-up data through July 2024 were reviewed. Data were obtained via the institutional electronic health record, and variables of interest closely mirrored those collected in the study by Akimoto et al. [6]. Variables of interest included demographic data (age, sex, and race), preoperative symptoms, whether patients underwent pre-resection embolization, tumor characteristics, such as location, volume, and World Health Organization (WHO) histologic grade, imaging characteristics (T2-weighted hyperintensity, cyst formation, vasogenic edema, and calcification), perioperative complications, extent of resection on postoperative imaging, post-resection length of stay (LOS), follow-up length, and whether patients experienced recurrence. Tumor location was denoted as convexity/non-convexity, supratentorial/infratentorial, and skull base/non-skull base, as classified in the study by Akimoto et al. [6]. Skull base meningiomas included anterior fossa, middle fossa, tentorial, cerebellopontine angle, foramen magnum, and petroclival meningiomas. “Other” locations included parasagittal, falcine, and ventricular. Imaging characteristics were collected by two of the authors (A.N. and B.L.) via blind assessment. Discrepancies in the classification of imaging characteristics were addressed through discussion. Recurrence was defined as radiographic progression either by an increase in tumor size or an increase in peritumoral edema resulting in clinical symptoms. Postoperative imaging was typically performed within three days of resection and was scheduled to be repeated at three months, six months, and one year post resection. Further surveillance imaging was typically performed annually thereafter unless patients experienced new neurologic symptoms.

Referral of patients for angiography with or without subsequent embolization was the operative surgeon’s decision. All embosurgeries occurred within two days of resection and were performed by one of three faculty members within the department of neurosurgery. The risks/benefits of liquid embolics, particle embolics, and coils were considered on an individual case-by-case basis. The choice of embolic agent was at the discretion of the surgeon performing embolization. Generally, liquid embolic was preferred if there were no dangerous anastomoses and if the microcatheter was able to be advanced distally into the tumor. Post-resection LOS calculation began the day of tumor resection. Informed, written consent was obtained from patients to undergo any and all procedures. As the time to recurrence analysis was censored if patients were lost to follow-up, no other exclusion criteria were applied.

Outcomes of interest were operative time, estimated blood loss, hemoglobin drop from pre-angiography or pre-resection to post-resection, incidence of blood transfusions, radiographic extent of resection, post-resection LOS, perioperative complication (cranial nerve deficit, stroke, hemorrhage, seizures, surgical site infection, and cerebrospinal fluid fistula/leak requiring re-operation), death within 90 days, tumor recurrence, and time to recurrence. Continuous variables included tumor volume, operative time, estimated blood loss, hemoglobin drop, LOS, time to recurrence, and follow-up length. Tumor volume was manually calculated using the ABC/2 method [12]. Age was transformed into a categorical variable and specified as <65 years or >65 years. This study was approved by the Institutional Review Board.

All statistical analyses were conducted using R version 4.4.3 (R Foundation for Statistical Computing, Vienna, Austria). Baseline characteristics and outcomes between the non-matched embolization and non-embolization groups were compared utilizing Mann-Whitney U tests and Fisher’s exact test for continuous and categorical variables, respectively. One-to-one propensity score matching was performed on patients with WHO grade 1 tumors with the MatchIt package in R on the basis of age, sex, presence of symptoms preoperatively, tumor location, tumor volume, T2 hyperintensity, cyst formation, vasogenic edema, and calcification [13]. A caliper target of 0.2 was used. For analysis of the matched groups, the Wilcoxon signed-rank test and Fisher’s exact test were utilized for continuous and categorical variables, respectively. If a patient was missing data for outcome variables, they were excluded from analysis for that particular outcome. Kaplan-Meier curves were generated for recurrence-free survival, and the log-rank test and Gehan-Wilcoxon test were employed to analyze the association of embolization with time to recurrence. Analysis was planned for the matched cohort, the unmatched WHO grade 1 meningioma-only cohort, and the unmatched cohort consisting of all patients. Cox proportional hazards regression was also utilized to identify whether covariates, including the matched covariates and race, were associated with time to recurrence. Patients were censored at the end of follow-up. A significance level of α = 0.05 was specified a priori for all statistical analyses.

Sensitivity analysis was performed to further evaluate the association between embolization and time to recurrence. All of the aforementioned covariates, including race, as well as the extent of resection (gross total versus subtotal resection), were included in Cox proportional hazards regression models for the matched and unmatched cohorts. Simpson grade was not included as a covariate due to published concerns of its applicability in modern neurosurgery [14].

## Results

**Figure 1:**
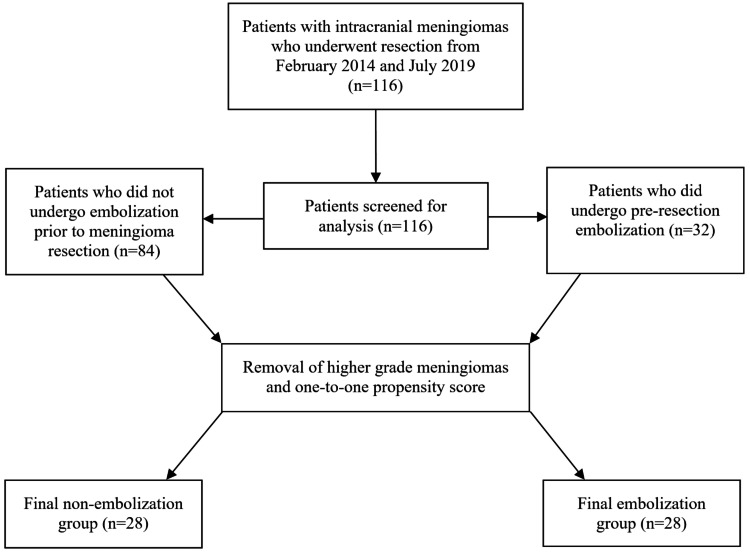
Flow diagram demonstrating the creation of the final study cohort.

A total of 116 patients met initial study criteria. Of the 116 patients, 39 (33.6%) underwent angiography, and 32 (27.6%) subsequently underwent embosurgery. The mean age was 61.0 + 1.2 years, and 76 (65.5%) were female. The mean follow-up time was 906.2 + 63.8 days (30.2 + 2.1 months) for the entire cohort. After exclusion of patients with WHO grade 2/3 meningiomas, propensity score matching resulted in 28 patients in each group. Standardized mean difference was <0.2 for each baseline variable. Cohort creation is depicted in Figure 1. The distribution of the variables utilized for propensity score matching can be found in Table 1. The distribution of race data is available in Table 2.

**Table 1:** Descriptive analysis of independent variables utilized for propensity score matching for both the unmatched and matched cohorts. ^a^ W for Mann-Whitney U (Wilcoxon rank sum) and odds ratio for Fisher’s exact test. ^b^ Variable not included in propensity score matching, so SMD not calculated. ^c^ Test statistic only available for 2 x 2 contingency tables in R. Abbreviations: mL, milliliter; SE, standard error; SMD, standardized mean difference; WHO, World Health Organization.

	Unmatched					Matched				
Variable	Embolized (n = 32)	Not Embolized (n = 84)	SMD	Test statistic^a^	p-value	Embolized (n = 28)	Not embolized (n = 28)	SMD	Test statistic^a^	p-value
Age (mean + SE)	59.6 + 1.9	61.5 + 1.4	-^b^	1496	.35	59.1 + 2.0	58.3 + 2.5	-^b^	399	.92
Age 65+ years	10	42	.39	0.46	.09	9	11	.15	0.74	.78
Female sex	17	59	.35	2.07	.13	15	16	.07	0.87	1.0
Asymptomatic	13	37	.07	0.87	.83	12	11	.07	1.16	1.0
Convexity tumor	10	27	.02	0.96	1.0	8	9	.08	0.85	1.0
Skull base tumor	13	27	.08	1.44	.39	10	10	< .001	1.00	1.0
Infratentorial tumor	3	9	.04	0.86	1.0	3	3	< .001	1.00	1.0
Other location	9	26	.06	0.87	.82	9	8	.08	1.18	1.0
T2 hyperintense	11	32	.08	0.85	.83	11	13	.14	0.75	.79
Vasogenic edema	24	52	.28	1.84	.20	21	20	.08	1.20	1.0
Cyst formation	2	6	.04	0.87	1.0	1	2	.16	0.49	1.0
Calcification	10	22	.11	1.28	.64	9	7	.16	1.41	.77
Volume in mL (mean + SE)	42.1 + 5.3	28.9 + 3.6	.42	873	.004	43.7 + 6.0	39.5 + 6.3	.13	421.5	.63
WHO grade			.02	-^c^	1.0			< .001	-^c^	1.0
1	28	72				28	28			
2	3	10				0	0			
3	1	2				0	0			

**Table 2:** Race data for the unmatched (all meningioma grades) and matched (only grade 1 meningiomas) cohorts. The test statistic is only available for 2 x 2 contingency tables in R.

	Unmatched			Matched		
	Embolized (n = 32)	Not embolized (n = 84)	p-value	Embolized (n = 28)	Not embolized (n = 28)	p-value
Race			.32			.06
Asian	1	1		1	1	
Black	8	16		7	1	
Hispanic	2	2		2	1	
White	21	65		18	25	

In the matched embolized group, surgical resection occurred, on average, 1.14 days after embolization (median = one day). Among patients who were embolized, 21 patients were embolized via the middle meningeal artery alone, one via the ascending pharyngeal artery alone, two via the occipital artery alone, one via a branch of the superficial temporal artery alone, and three via combinations of various external carotid artery (ECA) branches. Onyx Liquid Embolic System (Medtronic, Minneapolis, MN, USA) was used on its own in nine cases, in combination with coils in three cases, and in combination with particle embolic agents in one case. Coils were used solely in five cases and in combination with particle embolic agents in two cases. Particle embolic agents were used solely in eight cases.

After propensity score matching, most outcomes (except for postoperative LOS) were not significantly different between the embolized and non-embolized groups. The mean operative time was 350.0 + 34.4 minutes in the embolization group and 336.9 + 43.0 minutes in the non-embolization group. The mean estimated blood loss was 272.3 + 54.8 milliliters and 404.5 + 79.8 milliliters (P = 0.22) in the embolization and non-embolization groups, respectively. The mean hemoglobin drop was 2.1 + 0.3 grams/deciliter in the embolization group and 2.6 + 0.2 grams/deciliter in the non-embolization group (P = 0.08), with five perioperative transfusions (17.9%) occurring in the former and one (3.6%) occurring in the latter (P = 0.19). Reasons for transfusion in the embolized group included significant intraoperative estimated blood loss (n = 4) and low starting hemoglobin, which resulted in low postoperative hemoglobin (n = 1). The only transfusion in the non-embolized group was performed for significant intraoperative estimated blood loss. Immediate postoperative imaging (postoperative day one to three) was obtained for 26 patients in the non-embolization group, and 23 (88.5%) images were suggestive of gross total resection. All embolized patients underwent postoperative imaging, and gross total resection was achieved in 21 (75.0%) patients. Complications occurred in 7/28 (25.0%) and 6/28 (21.4%) of the embolization and non-embolization groups, respectively. The mean post-resection LOS was 9.9 + 1.3 days in the embolization group and 6.6 + 1.3 days for patients who did not undergo embolization (P=.002). Death within 90 days occurred in only one patient in the embolization group (3.6%). Of the surviving patients, recurrence was observed in one patient (3.6%) who did not undergo embolization and in six patients (22.2%) who were embolized. The mean time to recurrence in the embolization group was 539.7 days, and was 88.0 days for the one patient in the non-embolization group. The summary of these data can be found in Table 3.

**Table 3:** Study outcomes of meningioma resection of both the unmatched and propensity-scoring matched cohorts. ^a^ W for Mann-Whitney U (Wilcoxon rank sum) and odds ratio for Fisher’s exact test. ^b^ Test statistic only available for 2 x 2 contingency tables in R. ^c^ Excludes patients who expired within 90 days of surgery. ^d^ Excludes patients who did not have follow-up imaging beyond immediate postoperative imaging. Abbreviations: dL, deciliter; g, gram; mL, Inf, infinity; milliliter; SE, standard error.

	Unmatched				Matched			
	Embolized (n = 32)	Not embolized (n = 84)	Test statistic^a^	p-value	Embolized (n = 28)	Not embolized (n = 28)	Test statistic^a^	p-value
Mean operative time in minutes (mean + SE)	356.5 + 33.7	292.6 + 37.8	993.5	.03	350.0 + 34.4	336.9 + 43.0	348	.48
Estimated blood loss in mL (mean + SE)	257.0 + 51.8	303.5 + 69.1	1310.5	.84	272.3 + 54.8	404.5 + 79.8	466	.22
Hemoglobin drop in g/dL (mean + SE)	2.0 + 0.3	2.2 + 0.3	1509.5	.31	2.1 + 0.3	2.6 + 0.2	500	.08
Gross total resection (%)	25/32 (78.1%)	63/79 (79.7%)	0.91	1.0	21/28 (75.0%)	23/26 (88.5%)	0.40	.30
Transfusion (%)	6 (18.8%)	4 (4.8%)	4.54	.03	5 (17.9%)	1 (3.6%)	5.71	.19
Number of patients with any complication (%)	8 (25.0%)	26 (31.0%)	-^b^	.70	7 (25.0%)	6 (21.4%)	-^b^	1.0
Cranial nerve injury	2	5			2	3		
Stroke	2	10			2	2		
Hemorrhage	0	2			0	0		
Postoperative seizure	1	3			1	1		
Surgical site infection	2	2			1	1		
Cerebrospinal fluid leak	1	7			1	1		
Postoperative length of stay in days (mean + SE)	10.3 + 1.3	6.7 + 1.2	705.5	< .001	9.9 + 1.3	6.6 + 1.3	199.5	.002
Death within 90 days (%)	1 (3.1%)	3 (3.6%)	0.87	1.0	1 (3.6%)	0 (0%)	Inf	1.0
Mean follow-up time in months (mean + SE)^c^	1026.6 + 139.5	860.6 + 71.6	1118	.33	1058.4 + 156.9	1004.1 + 138.2	374.5	.96
Recurrence (%)^d^	7/31 (22.6%)	8/81 (9.9%)	2.63	.12	6/27 (22.2%)	1/28 (3.6%)	7.46	.05
Mean time to recurrence in days	616.7	671.0	29	.96	539.7	88.0	422.5	.62

**Figure 2:**
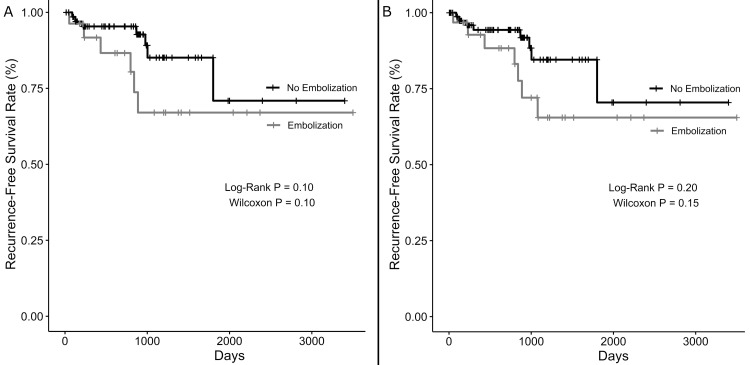
Kaplan-Meier curves demonstrating rates of recurrence-free survival over time, stratified by embolization status of (A) only patients with World Health Organization grade 1 meningiomas and (B) all study patients.

As only one event (recurrence) occurred in the non-embolized group of the matched cohort, survival analysis and Cox proportional hazards regression were not performed for the matched cohort. Log-rank test and Wilcoxon test did not demonstrate any association of embolization with time to recurrence, even when analyzed by all grade meningiomas and only WHO grade 1 meningiomas (Figure 2). In the unmatched WHO grade 1 meningioma-only cohort, Cox proportional hazards regression revealed that embolization was significantly associated with shorter time to recurrence (hazards ratio (HR): 10.0, 95% confidence interval (CI): 1.7-60.8, P = 0.01). Sensitivity analysis factoring in gross total resection demonstrated a similar association between embolization and increased chance of recurrence during the follow-up period (HR: 9.8, 95% CI: 1.4-68.8, P = 0.02). When the unmatched cohort of all WHO grade meningiomas was analyzed, Cox proportional hazards regression demonstrated that Black race (relative to non-Hispanic White race) was associated with shorter time to recurrence (HR: 3.4, 95% CI: 1.005-11.3, P = 0.049). Embolization was not significantly associated with time to recurrence in this analysis of all WHO grade meningiomas. No covariates were significant in the sensitivity analysis of all WHO grade meningioma patients.

## Discussion

The present study attempted to reproduce the benefits demonstrated by Akimoto et al. by examining the patients of a United States-based tertiary referral center [6]. The proportion of female patients, the overall complication rates, and recurrence rates were consistent with those of the published literature [6,15-17]. In this propensity score-matched retrospective cohort study, pre-resection meningioma embosurgery was not associated with differences in operative time, estimated blood loss, quantitative postoperative hemoglobin drop from preoperative levels, need for transfusion, incidence of postoperative complications, chance of achieving gross total resection, or death within 90 days, and was thus a safe procedure. Embolization was associated with increased post-resection LOS, and the average LOS of an embolized patient was 3.3 days longer than that of a non-embolized patient. Embolization was not associated with prolonged time to recurrence in survival analysis.

Rates of perioperative transfusion, complications, and death within 90 days were not significantly different between the groups, supporting that embolization is an overall safe procedure. There was a non-significant increased rate of complications in the embolization group, which may account, in part, for the increased post-resection LOS. However, as no benefits were observed, this study calls into question the utility of preoperative meningioma embolization. It is important to note that the lack of benefit is observed in the context of surgeon selection bias, as not all patients were referred for embolization. This possibly indicates that our institutional referrals for embolization are appropriate and that embolization makes the surgical resection risk for these patients on par with those of patients who were not referred.

In survival analysis of our unmatched cohorts of only WHO grade 1 meningiomas, embolization was associated with a shorter time to recurrence in both the main and sensitive analyses as opposed to a prolonged time to recurrence [6]. When higher-grade meningioma patients were included, embolization was not significantly associated with time to recurrence. However, a race difference was observed: Black patients were more likely to experience recurrence than White patients in the main analysis. No covariates were associated with time to recurrence in the sensitivity analysis of all meningioma patients. There are prior studies that demonstrated Black race is associated with meningioma recurrence as well as higher-grade meningiomas [18].

Akimoto et al. were able to demonstrate highly beneficial results with embolization [6]. We were unable to replicate these results in our patient cohort, which certainly represents a different population than that of the referenced study. Although location was described in the study, there was not enough granularity to determine whether the tumors that were less likely to embolize were clinoidal meningiomas with cavernous sinus invasion. Alternatively, perhaps there is another underlying characteristic of their patient population, such as genetic mutations that render the meningiomas more susceptible to embolization. Another possibility is that their technique, which they cited from Manaka et al., is a superior method for meningioma embolization [6,19]. The authors performed embolization with Embosphere particles (Nippon Kayaku, Tokyo, Japan) or polyvinyl alcohol, followed by coiling. The combination of particle embolization and coiling was only utilized in 2/32 of our patients. Furthermore, while embolization was associated with shorter time to recurrence in patients with WHO grade 1 meningiomas, when WHO grade 2 and 3 meningiomas were included, embolization was no longer significant. This may be due to surgeon selection factors such as a higher likelihood of embolization referral for greater complexity meningiomas, which are less likely to be successfully completely resected. It may also be due, in part, to tumor biology such as increased expression of hypoxia inducible factor-1 alpha and vascular endothelial growth factor [20]. Thus, while our results appear to disagree with those of Akimoto et al. [6], further investigations into embolization techniques and genetic or epigenetic differences are warranted.

This cohort study reports our institution’s experience with meningioma pre-resection embosurgery. While pre-resection embosurgery was safe, it was associated with a significantly prolonged LOS, raising the question of whether it was a particularly efficacious procedure. Furthermore, there are additional costs associated with procedure supplies, anesthesia time, and personnel required for embolization. Although propensity score matching was employed, there are likely other confounders present, and further research is needed to elucidate whether these factors demonstrate that meningioma embolization is efficacious or an unnecessary procedure.

There are several limitations of this study to note. This was a retrospective study, and surgeon selection bias may confound the results. Its generalizability may also thus be limited. Due to propensity score matching, the sample size was limited from 116 to 56, decreasing the power of the study’s univariable analysis. Although propensity score matching seeks to reduce confounding, there exists the possibility of unexplained confounders. Furthermore, certain outcomes, such as tumor recurrence, were not frequent. This study did not investigate differences between coils, liquid embolic agents, or particle embolic agents. Prospective randomized, controlled trials would provide the best insight into whether pre-resection meningioma embolization is efficacious.

## Conclusions

Pre-resection embolization of meningiomas is safe but was associated with an increased LOS by three days. Furthermore, embolization was not associated with prolonged time to recurrence in the study cohort. While propensity score matching was employed, selection bias inherently limits a non-randomized retrospective study of this nature, and further investigations are necessary to determine if particular patients may benefit from embolization or if particular techniques are superior for meningioma pre-resection embosurgery.
